# Oleic Acid Nanovesicles of Minoxidil for Enhanced Follicular Delivery

**DOI:** 10.3390/medicines5030103

**Published:** 2018-09-14

**Authors:** Pawan Kumar, Shailendra Kumar Singh, Vandana Handa, Himanshu Kathuria

**Affiliations:** 1 Department of Pharmaceutical sciences, Guru Jambheshwar University of science & Technology, Hisar 125001, India; pawansoni528@gmail.com; 2 School of Pharmacy, Krishna Institute of Engineering and Technology, Ghaziabad 201206, India; vandanahanda1979@gmail.com; 3 National University of Singapore, Singapore 117543, Singapore; a0120707@u.nus.edu

**Keywords:** ufasomes, oleic acid vesicle, skin deposition, minoxidil, hair follicle delivery

## Abstract

Current topical minoxidil (MXD) formulations involve an unpleasant organic solvent which causes patient incompliance in addition to side effects in some cases. Therefore, the objective of this work was to develop an MXD formulation providing enhanced follicular delivery and reduced side effects. Oleic acid, being a safer material, was utilized to prepare the nanovesicles, which were characterized for size, entrapment efficiency, polydispersity index (PDI), zeta potential, and morphology. The nanovesicles were incorporated into the emugel Sepineo^®^ P 600 (2% *w*/*v*) to provide better longer contact time with the scalp and improve physical stability. The formulation was evaluated for in vitro drug release, ex vivo drug permeation, and drug deposition studies. Follicular deposition of the vesicles was also evaluated using a differential tape stripping technique and elucidated using confocal microscopy. The optimum oleic acid vesicles measured particle size was 317 ± 4 nm, with high entrapment efficiency (69.08 ± 3.07%), narrow PDI (0.203 ± 0.01), and a negative zeta potential of −13.97 ± 0.451. The in vitro drug release showed the sustained release of MXD from vesicular gel. The skin permeation and deposition studies revealed superiority of the prepared MXD vesicular gel (0.2%) in terms of MXD deposition in the stratum corneum (SC) and remaining skin over MXD lotion (2%), with enhancement ratios of 3.0 and 4.0, respectively. The follicular deposition of MXD was 10-fold higher for vesicular gel than the control. Confocal microscopy also confirmed the higher absorption of rhodamine via vesicular gel into hair follicles as compared to the control. Overall, the current findings demonstrate the potential of oleic acid vesicles for effective targeted skin and follicular delivery of MXD.

## 1. Introduction

Minoxidil (MXD) was introduced clinically in the early 1970s as a hypertensive drug, and was later found to have side effects of hypertrichosis and regrowth of hair in bald males [1]. Currently, MXD and finasteride are the only topically approved agents so far by the Food and Drug Administration for the treatment of hair loss. The treatment is available as a solution (1% to 5% *w*/*v*) and as a 5% *w*/*v* foam formulation [2]. MXD has the ability to stimulate hair growth in both men and women [3,4]. It is believed that the topical MXD absorption affects the normal hair cycle by shortening of the telogen phase while enhancing the anagen phase of the hair cycle; however, the exact mechanisms are not yet clear [5]. Recently, another mechanism of action for MXD has also been reported [6]. The stratum corneum (SC) forms a significant barrier to limit the percutaneous absorption of drugs. Owing to the limited water solubility of MXD, current products generally contain organic solvents to increase the solubility of the formulation and penetration through skin [7]. However, these formulations show poor patient compliance as a result of several factors, including inconvenience of use, the necessity for multiple applications a day, a greasy effect after application, and in some cases severe adverse reactions such as contact dermatitis, facial hypertrichosis, scalp dryness, irritation, and burning, etc. [8,9,10,11]. A number of other drug delivery systems which vary in structure and compositions, such as niosomes [12], penetration-enhancing vesicles [4,13], foams [14,15], lipid nanocarriers [16,17,18], microbubbles [19], microneedles [19,20,21,22,23], inclusion complexes [2,24,25], lecithin microparticles [26], and effervescent formulations [27], have been developed to enhance the skin permeation of drugs including MXD. However, the limitations mentioned earlier have not yet been addressed. Hence, a novel formulation for the topical delivery of MXD is needed to combat these limitations. 

Recently, there has been increasing interest in nano-sized self-assembled bilayer vesicles to enhance drug penetration. Oleic acid (OA) has been in use for a long time as a penetration enhancer. Oleic acid vesicles (OAVs) have also been reported previously to enhance drug absorption via the skin [28,29,30]. It has been reported that formation of unsaturated fatty acid vesicles (ufasomes) is limited to a narrow pH (7–9) range, where fully ionized and unionized fatty acid carboxylic acid groups are arranged as bilayer vesicles [31]. OAVs have been used in studies as a carrier for a number of drugs via topical administration; however, none explored their potential for follicular drug delivery [32,33]. Ufasomes have been known to overcome the skin barrier, probably via lipid exchange between the SC layer and lipid carriers [23]. After penetrating through the SC into the dermis, they get washed out through the fusion of fatty acid in sebum around the hair shaft. Unlike other skin diseases, alopecia areata is specifically restricted to hair follicles. Therefore, it is essential to enhance drug absorption into the hair follicles for effective and safe treatment. Hence, the ideal formulation for hair follicle targeting should involve a composition and physical properties which can facilitate fusion of fatty acid in sebum. This makes fatty acid vesicles an attractive delivery system for targeted drug delivery to the hair follicles.

Hence, in the present work, self-assembled nanovesicles of OA and phosphatidylcholine were studied. The characterization of vesicle size, entrapment efficiency, and other physiochemical properties was performed. The vesicles were incorporated into the gel, and ex vivo skin permeation and deposition studies were conducted to determine the drug accumulation in different layers of skin. Confocal laser scanning microscopy (CLSM) was performed to visualize the drug permeation of vesicles in the excised rat skin.

## 2. Materials and Methods 

### 2.1. Materials

Minoxidil was available as a gift sample from Zee laboratories Ltd., Baddi, India. Sepineo™ P 600 (concentrated dispersion of acrylamide/sodium acryloyldimethyl taurate copolymer in isohexadecane) was received as a kind gift sample from Seppic (Seppic, Mumbai, India). In addition, 90% soya phosphatidylcholine (Phospholipon 90G^®^ OR PL90G) was received from Lipoid (Lipoid GmbH, Newark, NJ, USA). Rhodamine B (RB), Tween 80, and propylene glycol were purchased from Sigma-Aldrich, Mumbai, India. Oleic acid (OA) and dialysis membrane (molecular weight cut off 14,000 Da) were purchased from Hi-Media, Mumbai, India. All other chemicals were of analytical grade. 

### 2.2. Preparation of Vesicles

OAVs were prepared by the vortex shaking method [34]. Briefly, known quantities of MXD were added to different ratio of OAs and Phospholipon 90G^®^ (PL90G) (Table 1), which were dissolved in 2 mL of ethanol by shaking in a covered beaker. The homogeneous mixture was freeze-dried for 12 h to remove the residual organic solvent. Later, 10 mL of phosphate buffer saline (PBS, pH 7.4) were added into the beaker for film hydration with continuous vortexing at a temperature above the phase transition temperature of PL90G (60 ± 2 °C) to form cloudy suspension of OAVs. The resulting suspension was sonicated (Sonics & Materials, Newtown, CT, USA) for 15 min to reduce the vesicle size to nanometers. Later, the OAVs were extruded manually (Liposo Fast-Basic, Avestin, ON, Canada) 10 times via a polycarbonate filter (pore size 300 nm) to obtain uniform-sized vesicles. The developed MXD-OAVs were lyophilized with mannitol (5% *w*/*v*, as a cryoprotectant) using a lyophilizer (Labconco benchtop freeze-dryer system, Labconco Corporation, Kansas, MO, USA). Firstly, the suspension was pre-frozen at −80 °C for 12 h and subsequently lyophilized at a temperature of −25 °C for 24 h, followed by a secondary drying phase of 12 h at 20 °C. The finalized vesicle dispersion (0.2%) was filled in glass vials after lyophilization and stored in desiccator at 4 °C until further use. The RB-loaded OAVs were prepared using same composition and method used for MXD-OAVs, where the RB concentration was 0.1%. The control solution (MXD-Lotion) was prepared with a similar composition (propylene glycol/water/ethanol in 20:30:50 ratio) to that available on the market by dissolving 2% *w*/*v* of MXD. 

### 2.3. Vesicle Physical Characterization 

Dynamic light scattering (Zetasizer nano ZS90, Malvern Instruments, Malvern, UK) was utilized to measure the average particle size, zeta potential, and polydispersity index (PDI) of the prepared OAVs. The samples were diluted 10 times with distilled water. The zeta potential of MXD-OAV dispersions were measured under an electrical field of 40 V/cm. The measurements were conducted in triplicates at 25 °C using the automatic mode. The drug entrapment efficiency (%EE) was determined using a centrifugation technique. The dispersion was centrifuged (3K30, Sigma, Osterode am harz, Germany) at 15,000 rpm for 4 h at 4 °C. The supernatant was separated, and sediments were re-suspended in PBS. Later, the suspended vesicles were lysed using 0.2% *v*/*v* Triton X-100 and analyzed for the entrapped MXD content spectrophotometrically at λ_max_ 231 nm with a Shimadzu UV spectrophotometer (Shimadzu Corporation, Tokyo, Japan). The %EE was determined by dividing the entrapped drug in formulation divided by the total drug content of the formulation.

### 2.4. Differential Scanning Calorimetry (DSC) 

The thermal behavior of pure MXD, PL90G, mannitol (MNT), and MXD-OAVs was studied using TA Q100 DSC Instruments (TA Instruments, New Castle, DE, USA). Calibration of the heat flow scale was done before adding the samples for analysis. A small amount (5 mg) of each material (pure drug, PL90G, and lyophilized MXD-OAVs) was weighed in aluminum pans followed by crimping. The thermogram was recorded at a 20 mL/min nitrogen gas flow rate and a heating rate of 10 °C/min over a temperature range of 20 °C to 240 °C.

### 2.5. Thermogravimetric Analysis (TGA)

A thermoanalyzer, the TA SDT Q600 Instrument (TA Instruments, New Castle, DE, USA), was used to determine thermal properties of pure MXD, PL90G, and MXD-OAVs. The equipment was operated under a dynamic nitrogen gas atmosphere, with a temperature from 30 °C to 320 °C at 10 °C/min heating rate. The results were presented as percent weight loss with respect to temperature.

### 2.6. X-Ray Diffraction (XRD) Study

The diffractograms of MXD and powdered MXD-OAVs were recorded using a Bruker D8 ADVANCE diffractometer at 2*θ* between 10° and 70°. CuKα monochromatic radiation (λ = 1.5418°A) at 40 kV and 40 mA was used as the X-ray source to analyze the samples.

### 2.7. Transmission Electron Microscopy (TEM)

The shape and aggregation of prepared vesicles were examined using TEM (H-7500, Hitachi, Tokyo, Japan). One drop of sample was placed on a carbon-coated copper grid (300 mesh), and subsequently dried in open air for 10 min after negative staining with 2% phosphotungstic acid solution.

### 2.8. Preparation of Vesicular Gel 

The formulation of MXD and rhodamine B (RB)-loaded OAV gel was done by mixing Sepineo^®^ P600 (2% *w*/*v*) with a corresponding dispersion with continuous stirring at 500 rpm for 15 min. Care was taken during the mixing process to avoid air entrapment. The resultant gels (MXD-OAV-Gel and RB-OAV-Gel) were left for equilibration at room temperature (25 ± 1 °C) for 24 h. The pH of the prepared gel was determined using a digital pH meter (Sartorious PB11, USP, Sartorius Lab Instruments GmbH & Co. KG, Goettingen, Germany). The other details on preparation and characterization of gel have been previously reported [35].

### 2.9. In Vitro Drug Release 

The amount of MXD release from lipid vesicles was determined using a Franz diffusion cell method. Here, 200 mg of both MXD-OAV-Gel (0.2%) and MXD-Lotion (control) were applied on dialysis membrane with a molecular weight cut-off of 12,000–14,000 Da, mounted on a Franz diffusion cell. The control solution (MXD-Lotion) was prepared in similar composition to that available on the market by dissolving MXD (2%) in propylene glycol/water/ethanol in a 20:30:50 ratio. PBS (pH 7.4) and ethanol (70:30 *v*/*v*) were used as a dissolution media maintained at 32 ± 0.5 °C by a circulating water jacket. The exposed area of membrane for release was 0.785 cm^2^. The continuous stirring of the medium was ensured using a magnetic stirrer and magnetic bead. At predetermined time intervals, samples (1 mL) were withdrawn and replaced with equal amounts of fresh dissolution media. All samples were analyzed by a UV spectrophotometer at λ_max_ 231 nm and the cumulative % release of MXD was calculated [36]. 

### 2.10. Ex Vivo Skin Study 

#### 2.10.1. Skin Permeation

A Franz diffusion cell (diffusion area 2.0 ± 0.1 cm^2^) was used for this study. The experimental protocol was approved by the Institutional Animals Ethics Committee (registration number 0436; date of approval: 20 June 2016). The pig ear skin used for experiment was collected from ears obtained from local slaughter house. The skin was trimmed, washed under tap water, and dried with tissue paper. A skin specimen (3 × 3 cm) was cut and mounted on Franz diffusion cells in such a way that the SC faced donor compartments. The receptor compartment was filled with 6.0 mL of PBS pH 7.4 and ethanol (30% *v*/*v*) maintained at 37 ± 1 °C throughout the experiment under continuous stirring (500 rpm). After equilibrium, 200 mg of MXD-OAV-Gel (0.2%) and MXD-Lotion (2%) were applied to the donor compartment without any massage. For better simulation with patient use, no additional protection was implemented. At predetermined time intervals, aliquots (0.2 mL) were withdrawn from the receptor compartment for 24 h and were replaced with an equal volume of fresh receptor medium to ensure skin conditions. The aliquots were analyzed for MXD content by HPLC at λ_max_ 231 nm. 

#### 2.10.2. Drug Deposition

The differential stripping technique was used to measure the MXD content in hair follicles. After 24 h of the experiment, the skin was removed from Franz diffusion cells and placed on flat surface followed by cleaning with gauge pad soaked in water. The tape stripping was performed 15 times using adhesive tape (Transpore 3M surgical tape, 3M India Ltd., Gurugram, India) on the skin to remove the SC. The cynoacrylate skin biopsy was used to prepare the follicular cast, where a drop of super glue (Loctite Super Glue, Avon, OH, USA) was added on a glass slide and pressed on the surface of the stripped skin area. After total polymerization of the glue (~5 min), the glass slide was removed from skin with one quick movement. The super glue containing the hair follicles on glass slide was scraped off in a vial containing 2 mL of methanol for MXD extraction. The remaining skin and adhesive tapes were minced with a surgical sterile scalpel, added to separate tubes containing methanol, and grinded for 5 min followed by homogenization at 15,000 rpm for 10 min for MXD extraction. The resulting suspension was filtered using a 0.22-µm filter for HPLC analysis [37,38]. However, due to the difference in MXD content in applied formulations (i.e., vesicular gel has 10 times less drug content than lotion), the results were expressed as delivery efficiencies (*DE%*) (Equation (1)) as described in the below equation for a better comparison of delivery data.

(1) $$DE\%=\frac{Amount\;of\;drug\;delivered\;per\;unit\;area}{Amount\;of\;drug\;applied\;per\;unit\;area}$$

The follicle targeting factor (*FT*) (Equation (2)) was calculated to determine the targeting potential of prepared MXD-OAV-Gel. The equation given below was used to calculate the *FT* [27].

(2) $$FT=DC_{FC}/DC_{SC}$$

where *FT* is the targeting factor; *DC_FC_* is drug concentration in follicular casts; and *DC_SC_* is the drug concentration in the SC.

### 2.11. HPLC Method 

The reversed-phase adsorption chromatographic method was used to quantify the MXD by using HPLC (model LC-20AD, Shimadzu, Kyoto, Japan) coupled with a UV-visible detector set at 231 nm. The separation was achieved at following conditions: C18 column (5 µm, 250 mm × 4.6 mm) (Phenomenex, Macclesfield Cheshire, UK); 50 μL of injection volume; mobile phase consisting of methanol/water/glacial acetic acid (750/250/10, *v*/*v*/*v*, pH 3.0) and flow rate of 1.0 mL/ min. The lower limit of detection was 0.59 µg/mL, the lower limit of quantification was 1.99 µg/mL, and the linearity range was 2–12 µg/mL. 

### 2.12. Confocal Laser Scanning Microscopy 

The protocols for the experiment on albino rats were duly approved by the Institutional Animal Ethics Committee. The skin was prepared from excised skin from sacrificed rat followed by hair clipping, surgical removal of subcutaneous fat, and then, finally, washing of the skin with PBS. The skin piece was positioned on Franz diffusion cell to study the control (RB-Solution, 0.1% in OA) and test formulation (RB-OAV-Gel). After the end of the experiment (24 h), the full thickness of the exposed skin was directly studied under CLSM (TCS SP8, Leica, Wetzlar, Germany). The skin was scanned through the *Z*-axis of the microscope at increments of 5 µm. The RB excitation and emission wavelength were set to 515 nm and 525–635 nm, respectively.

### 2.13. Statistical Analysis of Data

Data analysis was carried out by analysis of variance (ANOVA) and Student’s *t*-test. Results were expressed as a mean ± standard deviation (SD). A statistically significant difference was determined at a minimal significance level of 0.05. 

## 3. Results and Discussion

### 3.1. Preparation and Characterization of Vesicles

MXD-OAVs were prepared with a constant amount of drug while varying the amount of the lipids (Table 1). It was found that the vortex shaking speed and time affected the size and uniformity of vesicles. The optimum speed of shaking and time were 100 rpm and 10 min, respectively, which were adequate to provide uniform size dispersion and to remove the organic solvent from dispersion. The addition of preheated PBS in the above formulation results in the ionization of half of carboxylic acid and transforms into ionized amphiphile(s) with a tendency to form typical dimers [39]. The probe sonication was done to convert the large vesicles into smaller size vesicles. The optimum sonication time was 15 min to obtain a homogeneous dispersion. The finalized formulation (OAV1) consisted of three parts of fatty acid and one part of PL90G, and was closely related to the stable liposomal formulation developed by other researchers [40,41,42]. 

The final MXD-OAVs (OAV1) showed a vesicle size of 317 ± 4 nm, PDI of 0.203 ± 0.006, zeta potential of −13.97 ± 0.451, and %EE of 69.08 ± 3.07. From Table 1, it is observed that when OA concentration was increased, vesicle size (317 nm to 377 nm) also increased while entrapment efficiency decreased (69.08 ± 3.07 to 58.85 ± 3.00). This result was well correlated with a report by Mittal et al. [42]. They also observed an increase in vesicle size and decrease in entrapment efficiency up to the optimum concentration of OA in vesicle formulation. The increase in the amount of PL90G increased the %EE of MXD. This could be attributed to the fact that unsaturated phospholipids are more flexible in nature, and thereby provide more space to retain MXD in lipid bilayer. Parmar reported similar observations for budesonide [32]. A narrow vesicle size distribution (~0.2) was obtained in this study, indicating the formation of a homogeneous dispersion. All three prepared samples of MXD-OAVs demonstrated a sufficiently negatively charged surface. In general, the optimum charged surface can provide a good physical stability of the formulations by creating electrostatic repulsion between the vesicles, which avoids the occurrence of vesicle aggregation or coalescence. Verma et al. reported on negatively charged clotrimazole OAVs [32]. Zakir et al. also showed a minimal increase in particle size of fluconazole-loaded OAVs (without incorporation into gel) over 30 days at 4 °C [33]. Therefore, the incorporation of OAVs into the gel will further improve the stability by decreasing the coalescence. 

### 3.2. DSC Analysis

**Figure 1:**
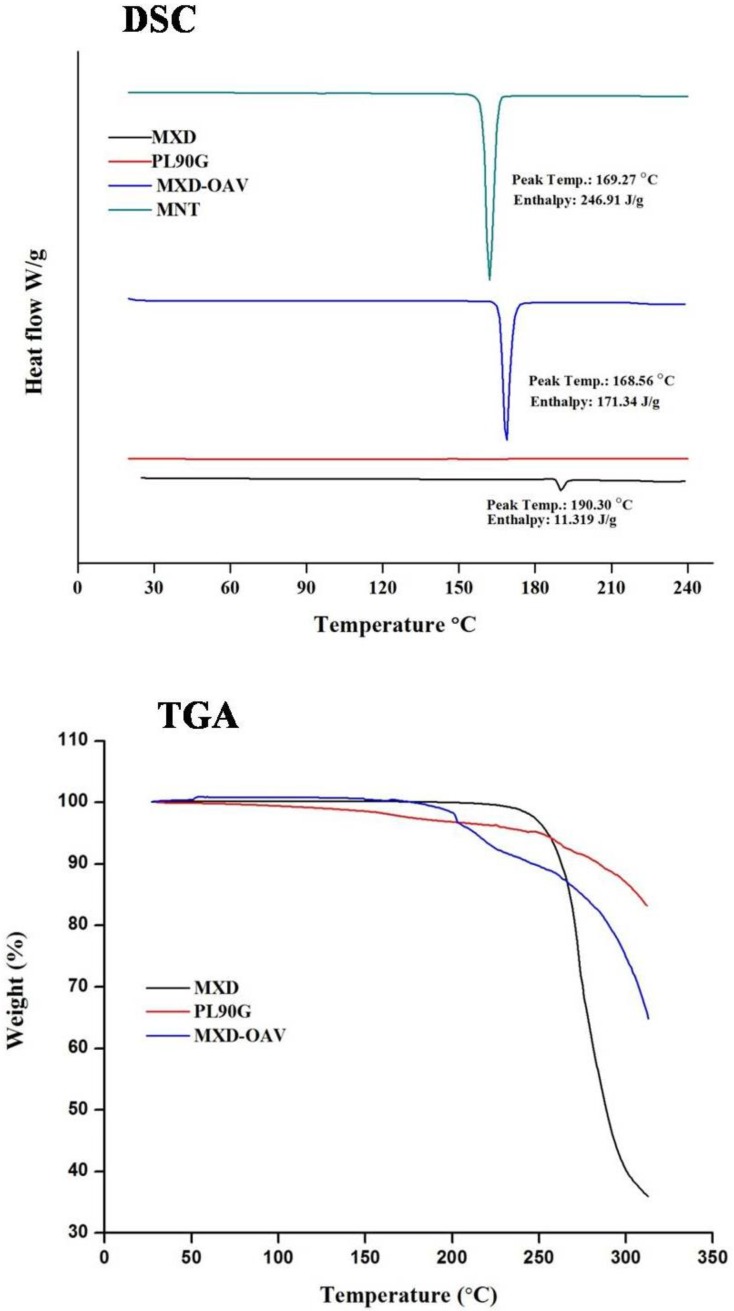
Differential scanning calorimetry (DSC) thermograms and thermogravimetric curves of MXD, 90% soya phosphatidylcholine (PL90G), mannitol (MNT), and MXD-OAVs. TGA: thermogravimetric analysis.

DSC analysis was conducted to investigate the amorphous and crystalline behavior of both drug and final formulation. The endotherm of MXD, PL90G, and the finalized formulation is given in Figure 1. The MXD in crystalline form was confirmed by small endothermic peak at 190.30 °C. While the DSC thermogram of PL90G revealed no sign of a thermal event, the temperature ranged from 20 °C to 240 °C. This can be attributed to presence of unsaturated phosphatidylcholines and the double bonds in the alkyl chains responsible for their loose packing in crystal lattice, which consequently lower the phase transition and melting temperatures below the room temperature [43]. The endothermic peak of MNT used as cryoprotectant was observed at 169.27 °C. The thermogram of the lyophilized MXD-OAVs did not show the endothermic peak for MXD around 190 °C indicating the molecular dispersion of drug inside the lipid matrix [44]. The endothermic peak observed at 168.56 °C belonged to MNT. The crystallinity of MNT was also confirmed by the XRD spectrum.

### 3.3. Thermogravimetric Analysis 

The TGA thermogram obtained with the MXD, PL90G, and drug-loaded vesicles samples is shown in Figure 1 The thermogram shows the degradation of MXD in the range of 248–304 °C, relative to the percentage weight loss of 88.32%. Similarly, PL90G showed a weight loss 16.8% in temperature range of 60–310 °C. The OAV formulation shows decomposition in two stages. At the first stage (below 180 °C), 7.52% weight loss was found corresponding to stoichiometric weight loss of the water molecules. Finally, a weight loss of 35.15% was observed in range of 262.5–310 °C, which can be attributed to degradation of other vesicle components.

### 3.4. XRD Analysis

**Figure 2:**
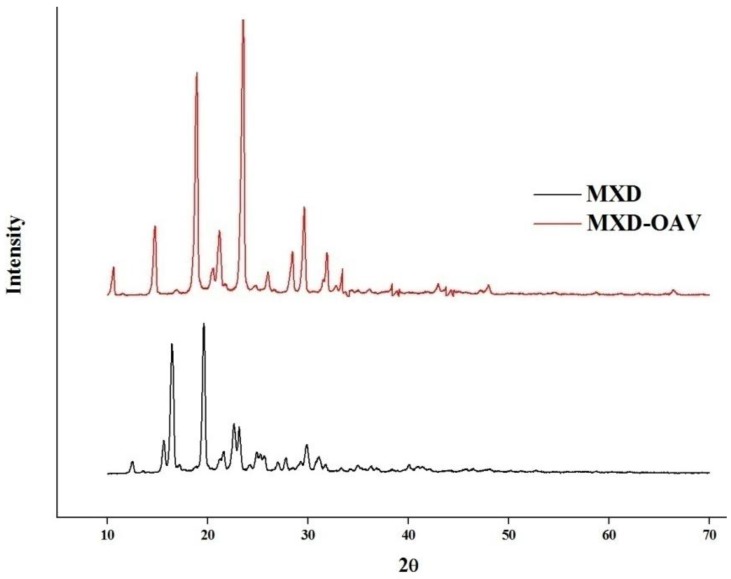
X-ray diffraction (XRD) spectrum of the drug and finalized formulation (OAV1).

The characteristic sharp peaks at 2*θ* values of 12.52°, 15.64°, 16.44°, 19.62°, 21.62°, 22.62°, and 23.12° were observed in the diffractogram of MXD (Figure 2), showing its crystalline nature. This was supported by DSC of MXD (Figure 1). These seven peaks were taken as a reference for comparison, and were not present in the diffractogram of the lyophilized MXD-OAVs within the respective positions and intensities. The results of the XRD determination confirmed that MXD was majorly in an amorphous form in the MXD-OAVs. Lyophilized MXD-OAVs showing characteristic peaks of MNT (at 2θ values of 10.6°, 14.7°, 23.4°, and 29.5°) confirmed our inference with respect to DSC results. Of note, XRD analysis of the PL90G and MNT was not performed. The PL90G was sticky in nature, while MNT XRD has been widely reported [44,45,46]. The reduction in crystallinity of MXD to majorly amorphous form of MXD in MSD-OAVs can be attributed to the solubilization effect of OA and phosphatidylcholines for MXD, leading to a majorly amorphous form after formulation. Punna et al. have also reported the reduction in crystallinity of raloxifene in a lipid matrix [46].

### 3.5. Morphological Study

**Figure 3:**
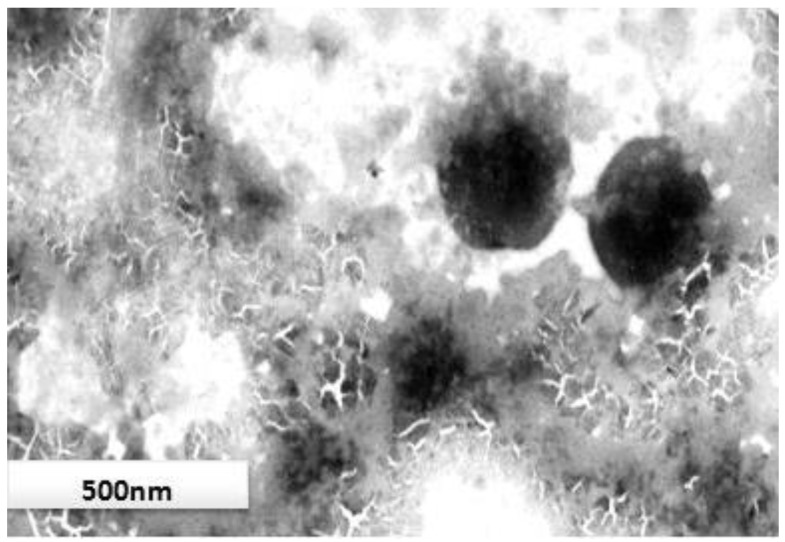
TEM image of the finalized formulation (OAV1) showing a spherical shape.

TEM analysis of finalized formulation (OAV1) showed that the vesicles were spherical in shape (Figure 3). However, difference in the vesicle size was observed by TEM analysis and Zetasizer, which could be attributed to dehydration of particles while preparing for TEM analysis and measurement of apparent diameter instead of actual diameter of the nanovesicle by Zetasizer [47]. The size distribution of OAV was better shown by the PDI obtained by the Zetasizer instead of TEM.

### 3.6. Formulation and Characterization of Gel

The prepared MXD-OAV dispersion showed vesicle aggregation and clear oil droplets on the surface of dispersion within one week of storage at room temperature. This could be attributed to the weak non-covalent bonding between the fatty acids and their ionized species resulting in the weak colloidal stability. Furthermore, the low residence time of vesicular dispersion also not ideal for inducing therapeutic effects. The incorporation of the finalized OAVs in the gel would improve its stability by preventing the vesicles’ physical aggregation and increasing contact time on the applied surface by enhancing adhesion [48]. The pH of the final formulation was 6.5, which was suitable for application to the skin. 

### 3.7. In Vitro Drug Release

**Figure 4:**
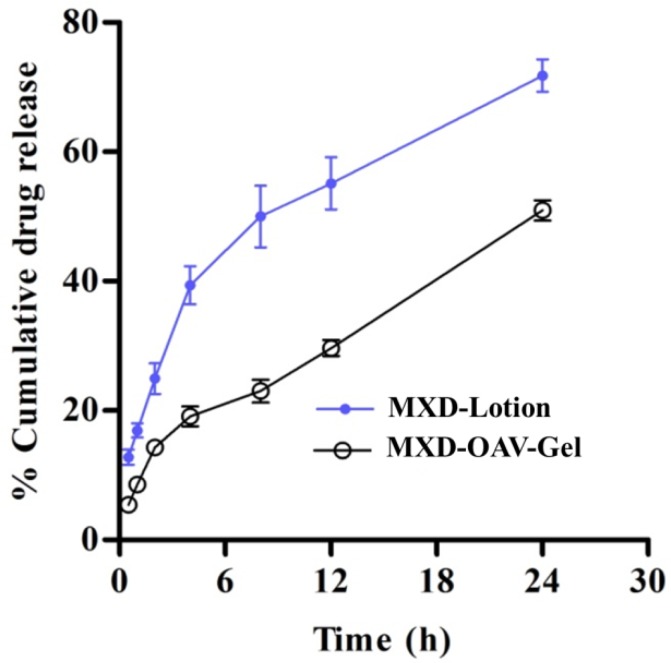
In vitro release of drug from MXD-Lotion and MXD-OAV-Gel. Values have been presented as mean ± SD (*n* = 3).

The drug release of vesicular gel was performed in order to evaluate the release profile. The MXD has very poor aqueous solubility in water; therefore ethanol (40% *v*/*v*) was added to release the medium to maintain skin condition. Figure 4 shows the comparative release of MXD-OAV-Gel and MXD-Lotion (control). The cumulative drug release from MXD-OAV-Gel (43.94 ± 1.56%) was found to be slower and more gradual than that of MXD-Lotion (71.78 ± 2.50%). The significantly different (*p* < 0.05) release rate can be attributed to the solubilizing effect of co-solvents such as propylene glycol and ethanol present in lotion that would have favored the quick release of drug in the dissolution medium. The MXD-OAV-Gel prolonged the MXD release; this could be due to the slower diffusion of the drug through polymeric matrix of the gel in addition to release from vesicles [49]. A similar finding was observed by Hussain, as they demonstrated the slower release of 5-flurouracil from liposomal gel as compared to solution [50].

### 3.8. Ex Vivo Skin Study

#### 3.8.1. Drug Permeation

**Figure 5:**
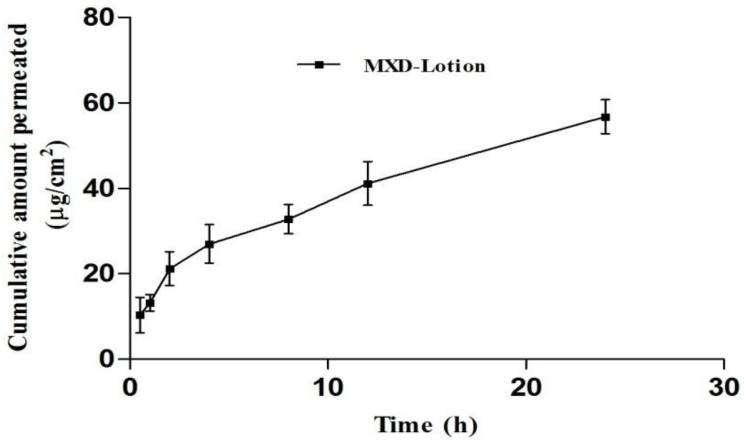
Cumulative amount of drug permeating through full-thickness pig ear skin from MXD-Lotion (mean ± SD, *n* = 3).

The permeation of MXD across full-thickness pig ear skin was performed using a Franz diffusion cell from MXD-Lotion (2%) and MXD-OAV-Gel (0.2%). The skin permeation is shown in Figure 5. The amount of drug permeated from MXD-Lotion was 56.78 ± 3.95 µg/cm^2^; whereas no trace of drug was found in receptor medium after the application of MXD-OAV-Gel. This suggested that the systemic exposure and its related side effects of MXD can be avoided. This could be attributed to the difference in mechanism of drug permeation across the skin from vesicular gel and lotion. The high delivery of the MXD-Lotion was expected due to the solvent system (ethanol and propylene glycol). The ethanol and propylene glycol act as penetration enhancers, which could alter thermodynamic activity of the drug in the vehicle when they permeate through the skin, consequently facilitating uptake of drug through skin which would in turn modify the driving force for diffusion [51]. In addition, the rapid evaporation of ethanol from drug lotion in the donor compartment would have increased the drug concentration to a supersaturated state with greater driving force for drug absorption [52]. In accordance with the result of previous studies, the skin permeability of drugs was significantly higher from OAV dispersion than control, possibly by a deformable characteristic of OAVs that allows the drug molecules to easily penetrate across the skin [33,53]. The potential difference behind disagreement of present and previous results could be explained by the presence of gelling agent in the final formulation (MXD-OAV-Gel), which released the drug slowly when compared with the dispersion, accounting for the diffusion lag time. 

#### 3.8.2. Drug Deposition

**Figure 6:**
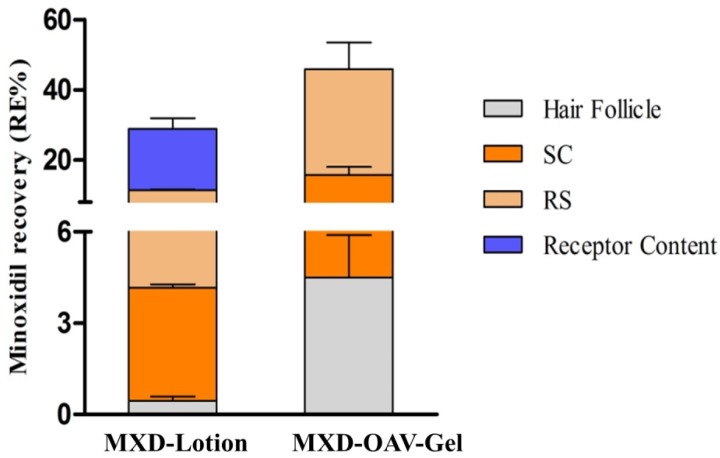
MXD retained in the stratum corneum (SC), hair follicles, and remaining in skin (RS), as well as receptor content after 24 h of skin permeation for MXD-Lotion (Control) and MXD-OAV-Gel (mean ± SD, *n* = 3).

**Figure 7:**
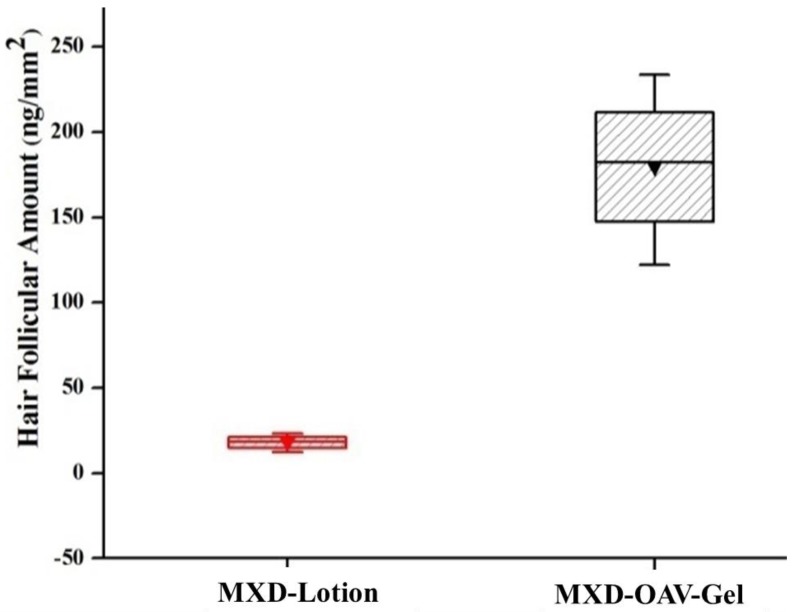
Amount of MXD recovered from hair follicles after cyanoacrylate biopsies (mean ± SD, *n* = 3).

The ex vivo skin retention studies were performed to measure the depot effect of vesicular gel in various compartments of skin (SC, hair follicles and remaining skin). For effective management of alopecia areata, the dosage form should deliver the drug inside hair follicles. The comparative amount of drug accumulation in skin compartments from MXD-OAV-Gel and MXD-Lotion is shown in Figure 6. The proportions of MXD recovered from SC and follicular casts and remaining in the skin after the application of MXD-Lotion were 3.71 ± 0.11%, 0.45 ± 0.13%, and 7.24 ± 0.21%, while for MXD-OAV-Gel the proportions were 11.22 ± 2.38%, 4.48 ± 1.39%, and 30.13 ± 7.60%, respectively. The MXD deposition was significantly higher (three-fold) in SC for vesicular gel as compared to MXD-Lotion (*p* < 0.05). The enhanced MXD deposition for OAV could be attributed to deformable characteristics that allowed the drug to flexibly and easily penetrate into the skin [54,55]. In addition, as OA has been well known as a penetration enhancer; it can interacts with the SC and modifies the highly packed intercellular lipids, thus facilitating permeation of drugs [56]. The MXD accumulation from MXD-Lotion was low, which could be due to higher amount of drug permeating through the skin instead of getting retained in the skin (Figure 6). The MXD accumulation in the skin was four times higher for vesicular gel than MXD-Lotion. The presence of PL90G (a major component of OAVs) was expected to enhance the fluidization of lipids in the skin, leading to drug absorption to the deeper skin layers [57]. This finding was supported by previous study of Lee et al. [26], who showed improved drug deposition in dermal layers from lecithin microparticles. The recovery of MXD from the hair follicle cast was done by a differential stripping technique and results are shown in Figure 7. The MXD-OAV-Gel yielded 10-fold superior depositions to the follicular cast as compared to MXD-Lotion (*p* < 0.05; one-way ANOVA with post hoc comparison by Tukey’s test), confirming that OAVs could target the hair follicles. In fact, a significantly (*p* < 0.05) higher FT value was observed for MXD-OAV-Gel (FT_MXD-OAV-Gel_ = 0.40) in comparison to solution control (FT_MXD-Lotion_ = 0.12). The flexible nature of OAVs as reported in the literature could be an attribute for gaining access to appendages (hair follicle and pilosebaceous unit) more readily, with high compatibility with the sebum to form a depot within the hair follicles by easy fusion with them [58].

### 3.9. Confocal Laser Scanning Microscopy

**Figure 8:**
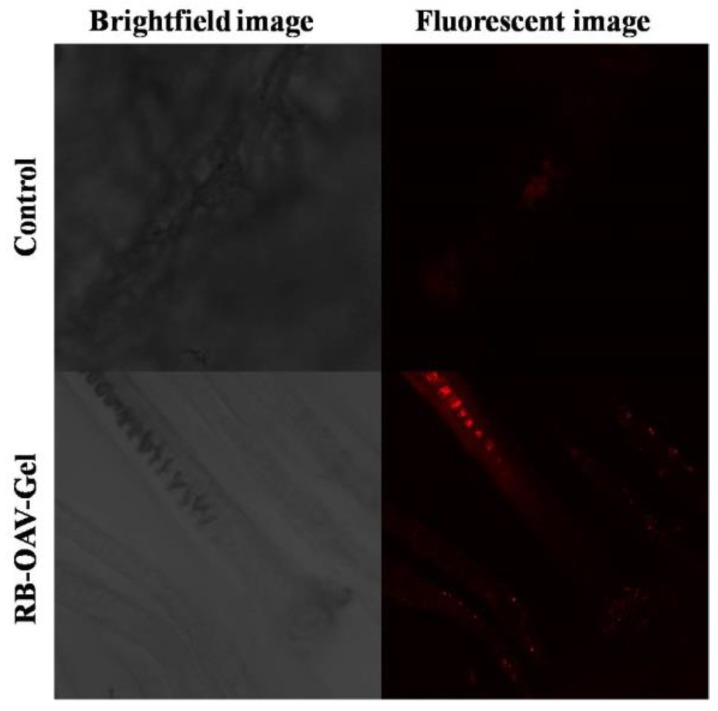
Confocal laser scanning microscopy images: XY-plane images of porcine skin after 24 h of application of the control and RB-OAV-Gel. Right panel and left panel show bright field and fluorescence images for the control and RB-OAV-gel, respectively. The fluorescent intensity was higher for RB-OAV-gel, and the stained hair follicle for the same is visible. RB: rhodamine B.

The distribution and penetration depth of RB via RB-OAV-Gel and RB-Solution in the hair follicles were visualized by CLSM (Figure 8). Rhodamine B has log P of 2.37 and MXD has log P of 1.24. The RB was chosen as control as it has lipophilic nature with closer log P to MXD. The other factors such as molecular weight can also affect the skin absorption; however, the log P has been reported as most important factor. The extent of permeation for the RB and MXD may not be same but the route of absorption will remain the same due to closer lipophilicity. The higher distribution of RB in the hair follicles was clearly evident in skin treated with RB-OAV-Gel as compared to RB-Solution (control) in terms of florescence intensity. The study ratifies the deeper penetration of vesicular system leading to higher drug distribution around the hair follicles [58].

## 4. Conclusions

The study demonstrates the potential application of OAVs that can self-assemble to form nano-sized vesicles encapsulating MXD. The OAVs can improve the physiochemical attributes of MXD, enhancing skin and follicular deposition. The MXD demonstrated spherical morphology, better drug entrapment, desired vesicle size, and considerable enhancement in MXD delivery. In the future, in vivo studies with suitable animal models will be essential to support this efficacy. Further, pharmacological and clinical studies will be essential for clinical applications. Overall, our findings have paved the way for MXD vesicular gel for topical applications potentially with better efficacy, safety, and patient compliance. 

## Figures and Tables

**Table 1:** Different formulation of oleic acid vesicles (OAVs) for optimization of the final formulation. MXD: minoxidil; PDI: polydispersity index; %EE: entrapment efficiency; OA: oleic acid.

Code	MXD (mg)	Ratio of (OA:PL90G)	Vesicle size (nm)	PDI	Zeta Potential (mV)	%EE
**OAV1**	20	3.0:1.0	317 ± 4	0.203 ± 0.016	−13.97 ± 0.451	69.08 ± 3.07
**OAV2**	20	3.5:1.0	377 ± 5	0.293 ± 0.088	−14.70 ± 0.579	58.85 ± 3.00
**OAV3**	20	3.5:2.0	589 ± 6	0.331 ± 0.110	−13.64 ± 0.526	71.75 ± 3.11
